# Genetic variations in *NF-κB* were associated with the susceptibility to hepatitis C virus infection among Chinese high-risk population

**DOI:** 10.1038/s41598-017-18463-y

**Published:** 2018-01-08

**Authors:** Ting Tian, Jie Wang, Peng Huang, Jun Li, Rongbin Yu, Haozhi Fan, Xueshan Xia, Yaping Han, Yun Zhang, Ming Yue

**Affiliations:** 1 Department of Epidemiology and Biostatistics, School of Public Health, Nanjing Medical University, Jiangsu, China; 2 Institute of Epidemiology and Microbiology, Huadong Research Institute for Medicine and Biotechnics, Jiangsu, China; 3 School of Nursing, Nanjing Medical University, Jiangsu, China; 4 Department of Infectious Diseases, The First Affiliated Hospital of Nanjing Medical University, Jiangsu, China; 5 Faculty of Life Science and Technology, Kunming University of Science and Technology, Kunming, China

## Abstract

Polymorphisms within *NF-κB* pathway genes may be linked to hepatitis C virus (HCV) infection susceptibility and outcomes. We investigated the associations between single nucleotide polymorphisms (SNPs) in NF-κB and the susceptibility as well as resolution of HCV infection. A Chinese population, including 1125 uninfected control cases, 558 cases with spontaneous viral clearance and 898 cases with persistent HCV infection, was genotyped for four SNPs (rs11820062, rs230530, rs1056890 and rs3774963) using a TaqMan assay. Our logistic analyses indicate that the subjects carrying RelA rs11820062 A allele had a significantly increased risk of HCV susceptibility (*P*_*Bonferroni*_ < 0.003125 in a dominant or additive model). In stratified analysis, the increased risk associated with rs11820062 A allele on HCV susceptibility remained in some case subgroups. This study demonstrates that a genetic variant involved in the *NF-κB* pathway gene (rs11820062 A allele) is associated with an increased HCV susceptibility within a high-risk Chinese population.

## Introduction

More than 185 million people are estimated to be chronically infected with hepatitis C virus (HCV). In China, the prevalence of HCV is approximately 1.6% of the population^1,2^. Over 70% of HCV infected patients who fail to eliminate the virus go on to develop chronic hepatitis C (CHC), cirrhosis and hepatocellular carcinoma^3^. Variable HCV infection outcomes are related to both host genetic factors and virological factors. Recently, novel direct-acting antiviral agents (DAAs) have dramatically risen HCV cure rates to 95% and have served as alternatives to PEGylated interferon (IFN) plus ribavirin therapy for chronic hepatitis C^4,5^. However, the efficacy and safety of DAAs in real-world settings are not well understood. Genetic factors related to the occurrence and clearance of HCV infection need to be explored.

Nuclear factor κ-light-chain-enhancer of activated B cells (NF-κB) exists in almost all mammalian cell types, and controls the transcription of many target genes by binding κB response elements. Thus activated, it can influence cell differentiation, apoptosis, inflammation processes and immune response^6^. NF-κB is not a single transcription factor but rather a human protein family containing five members, including NF-κB1(p50), NF-κB2 (p52), RelA (p65), RelB, c-Rel^6,7^. These members can form several kinds of homodimers or heterodimers. In unstimulated cells, inactive NF-κB proteins exist in the cytoplasm in association with inhibitors of κB (IκB), including IκBα, IκBβ, IκBγ (p105), IκBδ (p100), IκBε and Bcl-3. Different dimers of NF-κB proteins can be activated in different ways depending on their association with IκBα or IκBβ, in which IκB is prone to phosphorylation and degradation^6–8^. The predominant activated form of NF-κB is a p50/p65 dimer that is associated with IκBα^6^. In the canonical pathway, after stimulation with a large number of NF-κB inducers, IκBα is phosphorylated by IκB kinase (IKK) at serines 32 and 36 and then becomes ubiquitylated by a specific E3 ligase, followed by the degradation by the 26S proteasome^6^. The released NF-κB dimer can subsequently translocate into the nucleus where its target gene is activated by binding to gene promoters with high affinity to NF-κB elements^6^.

NF-κB activity is necessary for both innate and adaptive immunity. It can be induced by a variety of stimulants such as bacterial and viral antigens, cytokines, oxidative stress, and triggers the transcription of many inflammatory mediators, including chemokines, pro-inflammatory cytokines, and adhesion molecules^6,9,10^. Consequently, aberrant activation or dysregulation of *NF-κB* signaling pathway genes contributes to the development of some autoimmune diseases, inflammation, and malignant disorders^11^; it is also involved with viral evasion and subversion with some types of infections via the biphasic regulation of NF-κB activity^12^.

Single nucleotide polymorphisms (SNPs) in the *NF-κB* signaling pathway genes have been associated with many diseases. SNP rs11820062 in *RelA* is not only significantly associated with kidney function and chronic kidney disease susceptibility^13^, but also with susceptibility to schizophrenia and may affect androgen receptor transcription factor (TF) binding^14^. Previous studies have also identified SNP rs230530 in *NF-κB1* as associated with liver cancer^15^ and alcohol addiction^16^. Furthermore, *NFκB*2 rs1056890 has been related to the development of multiple myeloma and response to bortezomib therapy^17^, and related to the inflammatory response among patients with secondary lymphedema following breast cancer surgery^18^ as well as other immune-related diseases^19,20^.

Given these findings about the critical role of *NF-κB* in the host immune response against infection, any genetic variation in *NF-κB* signaling pathway genes may affect the progression and outcomes of HCV infection. However, no research has addressed the association between the *NF-κB* genetic polymorphisms and HCV infection. Thus, this study aims to examine the relationships between *NF-κB* signaling pathway genes SNP rs1056890, rs11820062, rs230530 and rs3774963, and HCV infection outcomes within a high-risk Chinese population.

## Materials and Methods

### Ethics statement

The study was performed in accordance with the World Medical Association Declaration of Helsinki on ethical principles in medical research involving human subjects, and was approved by the medical ethics committee of Nanjing Medical University. All study subjects provided signed written informed consent.

### Study subjects

A total of 2581 subjects were recruited between October 2008 and May 2015 including 816 hemodialysis (HD) subjects from nine hospital hemodialysis centers in southern China, 510 intravenous drug users from Nanjing Compulsory Detoxification Center and 1255 paid blood donors from six villages within Zhenjiang City. These paid blood donors had become infected with HCV when they engaged in paid blood donation multiple times, some as often as 100 times or more. Subjects were excluded if they were co-infected with hepatitis B virus or human immunodeficiency virus (HIV), suffered from other liver diseases (including autoimmune, alcoholic or metabolic liver diseases), or were treated with any antiviral medications before or during the trial. All subjects were divided into three groups for further analysis: the HCV-uninfected controls (Group A) were subjects seronegative for anti-HCV and HCV-RNA; those in the spontaneous clearance group (Group B) were subjects with seropositive anti-HCV and seronegative HCV-RNA; and those in the persistent HCV infection group (Group C) were subjects with seropositive anti-HCV and HCV RNA. Group B and Group C were combined as the HCV infected group. All serological results were confirmed by three separate experiments within the 6 month follow-up period. The control subjects in group A were matched by age, gender and the village of recruitment (5-year intervals) with infected subjects belonging to group B or group C.

An interview was carried out with each participant, and a structured questionnaire was administered by well-trained interviewers to collect demographic information, an environmental exposure history and a medical history of HCV infection. Quality control procedures were established to guarantee the reliability of all data obtained.

### Viral testing

A 10 mL venous blood sample was collected from each participant after the interview. White blood cells were isolated by centrifugation and subsequently stored at −80 °C until use. Anti-HCV antibodies were tested using a third-generation enzyme-linked immunosorbent assay (ELISA: Diagnostic Kit for Antibody to HCV 3.0 ELISA, Intec Products Inc, Xiamen, China) according to the manufacturer’s instructions. HCV RNA was extracted from patient serum using Trizol LS Reagent (Takara Biotech, Tokyo, Japan), and reverse transcription PCR (Takara Biotech) was performed. The Murex HCV Serotyping 1–6 Assay ELISA (Abbott, Wiesbaden, Germany) was used to determine the type-specific antibodies of various HCV genotypes^21^.

### SNPs selection

Tag SNPs (rs230530 and rs3774963) were selected using Haploview software (version 4.2; Broad Institute, Cambridge, MA, USA) based on the linkage disequilibrium (LD) data of HapMap Phase II CHB (Chinese in Beijing) obtained from the HapMap database (http://www.hapmap.org/) or the 1000 Genomes Project database (http://www.1000genomes.org/). We used minor allele frequency (MAF) > 5% as the criteria among the Chinese Han population. Considering the potential regulatory effects of adjacent sequences, 2000 bp upstream and downstream of NF-κB gene transcription initiation sites were included in the analysis. SNPs located on 5′-UTR, 5′ flanking region (rs11820062), 3′-UTR (rs1056890), and exons with missense substitutions were considered, and combined with SNPs with some connection to the liver^15,16^ or or immune-related disorders^13,14,17–20^ reported in the literature. Utilizing the above strategies, four candidate SNPs, rs1056890, rs11820062, rs230530 and rs3774963, were selected for further study.

### Genotyping Assays

Protease K digestion was used to extract genomic DNA from subject peripheral blood leukocytes, followed by phenol–chloroform extraction and ethanol precipitation. Genotyping of the four SNPs was performed with a TaqMan allelic discrimination assay on an ABI 7900HT Real-Time PCR System (Applied Biosystems, Foster City, CA, USA). The primers and probe sequences for selected SNPs are shown in Table 1. The technicians who performed the genotyping were blinded to subject clinical data. For quality confirmation, two negative controls were set in each 384-well plate, and a 100% concordance was achieved in 10% random samples. The success rates of genotyping for the selected four SNPs were all above 95%. Samples that failed genotyping were excluded in the statistical analyses.

**Table 1:** Probes and primers used for *NF-κB* SNP TaqMan assay.

SNPs (genotype)	Gene	Region	MAF^a/b^	TaqMan-MGB probe/primers sequences
rs11820062 G > A	RelA	Intron 1 or nearGene-5	0.392/0.427	Probe-G: FAM-TCCCTCAGTTTTC-MGB
				Probe-A: VIC-TCCCTCAATTTTC-MGB
				Forward primer: CTTGACTCAGTTTCCCTCCACAC
				Reverse primer: GAGGGAAAACGGGGTAAGGAATC
rs230530 A > G	NF-κB1	Intron 3	0.473/0.476	Probe-A: FAM-CAAACATCTTAATTTACATTC-MGB
				Probe-G: HEX-AAACATCTTAATTTGCATTC-MGB
				Forward primer: AAAATGGACATACAAGCATTCTCCT
				Reverse primer: TGCAATAAATAAAGGCATATGGTGGT
rs3774963 G > C	NF-κB1	Intron 15	0.377/0.409	Probe-G: FAM-ATGTTCGACTCCCAC-MGB
				Probe-C: HEX-ATGTTCCACTCCCAC-MGB
				Forward primer: TGGAAGGCATGGTGTTTGG
				Reverse primer: TGTGACTGCTCCAGCCCATA
rs1056890 C > T	NF-κB2	3′-UTR	0.196/0.171	Probe-C: FAM-CACCTCCGAGAGC-MGB
				Probe-T: VIC-CACCTCTGAGAGCC-MGB
				Forward primer: TGGGCCTCAGGAGCCTAG
				Reverse primer: ATCAAAAGTTCAGGGGCGCTAG

MAF: minor allele frequency.

^a^Minor allele frequencies in control group.

^b^Minor allele frequencies from HapMap of Han Chinese in Beijing, China (CHB) (dbSNP, build128; available at http://www.ncbi.nlm.nih.gov/SNP/).

### *In silico* analysis

The function of SNPs in genes was predicted using the bioinformatics tool SNP function prediction online server (SNPinfo; http://snpinfo.niehs.nih.gov/). To detect and characterize the influence of the polymorphism sites on the secondary structure of genes, RNA secondary structures were predicted using the Vienna RNA Web Servers based on the latest Vienna RNA Package (Version 2.3.1) (http://rna.tbi.univie.ac.at/cgi-bin/RNAWebSuite/RNAfold.cgi). Secondary structures with the lowest free energies of minimum free energy (MFE) and centroid secondary structures (a structure with minimal base pair distance) for wild-type and mutant-type mRNA sequences were calculated and compared. Moreover, Human Splicing Finder (http://www.umd.be/HSF/) was used to identify possible splicing signal in sequences containing the SNP sites.

### Statistical analysis

χ^2^-test, Kruskal–Wallis test or one-way analysis of variance were used to appropriately analyze the distribution general demographic, clinical, virological features, and genotype frequencies among the three groups. Hardy–Weinberg equilibrium (HWE) for each SNP was estimated by χ^2^ goodness of fit test among controls. LD parameters (r^2^ and D′) were calculated using Haploview software. The associations between SNPs and the HCV infection outcomes were estimated by calculating the odds ratios (ORs) and 95% confidence intervals (CI), adjusted for age, gender and route of infection using binary logistic regression models. A *P*-value < 0.05 in a two-sided test was considered statistically significant. Bonferroni correction was used to correct for multiple comparisons. All statistical analyses were carried out with the Statistical Package for the Social Sciences (SPSS: version 20.0; SPSS Institute, Chicago, IL, USA) and Statistical Analysis System software (SAS: version 9.1.3; SAS Institute, Cary, NC, USA).

## Results

### Demographic and selected variables of participants

The demographic and other selected characteristics for a total of 2581 subjects are summarized in Table 2. There were no significant differences in the distribution of age or gender among the three groups, while ALT, AST, routes of infection and HCV genotype (all *P* < 0.001) showed significant differences.

**Table 2:** Demographic and clinical characteristics of control, HCV spontaneous clearance and persistent infection populations.

Variables	Group A (%)	Group B (%)	Group C (%)	*P* value
	n = 1125	n = 558	n = 898	
Age (mean ± s.d.)	49.54 ± 14.475	49.01 ± 13.541	49.73 ± 10.959	0.588^a^
Gender				0.520^b^
Male	422(37.5)	225(40.4)	341(38.1)	
Female	702(62.5)	332(59.6)	553(61.9)	
ALT(median(IQR),U/L)	19.12(8.00,21.00)	41.28(16.00,41.25)	49.05(21.00,59.25)	<0.001^c^
AST(median(IQR),U/L)	22.02(12.00,25.00)	38.21(20.75,40.00)	45.33(25.00.53.5)	<0.001^c^
Routes of infection				<0.001^b^
HD	631	101	84	
Drug use	198	162	150	
Blood donation	296	295	664	
HCV genotype				<0.001^b^
Genotype 1	—	141	243	
Genotype non-1	—	82	38	
Mixed genotype	—	42	235	

Abbreviations: ALT, alanine transaminase; AST, aspartate aminotransferase; ANOVA, analysis of variance; HCV, hepatitis C virus; HD, hemodialysis; IQR, interquartile range.

Group A: control group; Group B: spontaneous clearance group; Group C: persistent infection group.

^a^ *P* value of one-way ANOVA among three groups.

^b^ *P* value of χ^2^-test among three/two groups.

^c^ *P* value of Kruskal–Wallis test among three/two groups.

All four SNPs’ allele distributions in the control group were in accordance with the expectations of the Hardy–Weinberg equilibrium. Group A was considered as the control group when compared with group B + C (group A: *P* = 0.834 for rs1056890, *P* = 0.179 for rs11820062, *P* = 0.063 for rs230530, *P* = 0.303 for rs3774963) and group B was considered as the control group when compared to group C (group B: *P* = 0.512 for rs1056890, *P* = 0.479 for rs11820062, *P* = 0.366 for rs230530, *P* = 0.579 for rs3774963).

### Association between *NF-κB* genes polymorphisms and the susceptibility to HCV infection

The distributions of genotypes rs1056890, rs11820062, rs230530 and rs3774963 among the control group, spontaneous clearance group, and persistent HCV infection group are shown in Table 3. Three genetic models, including additive, dominant and recessive models, were used to analyze the association between each SNP and susceptibility to HCV infection. After adjusting for gender, age, and the route of infection, the results of logistic regression analysis demonstrated that individuals carrying the rs11820062 AA genotype had a significantly higher risk of HCV infection (*P* = 0.002) after Bonferroni correction at the level 0.05/16. In the additive and dominant statistical models, the relationship between the rs11820062 A allele and susceptibility to HCV infection remained statistically significant after Bonferroni correction (*P* = 0.002 and *P* = 0.003, respectively).

**Table 3:** Genotypic distribution of *NF-κB* signaling pathway genes among control, spontaneous clearance and persistent infection group.

SNPs(genotype)	Group A	Group B	Group C	Group (B + C)/Group A		Group B/Group C	
	n(%)	n(%)	n(%)	OR(95% CI)^a^	*P* ^a^	OR(95% CI)^b^	*P* ^b^
rs1056890							
CC	724(64.8)	369(66.4)	608(68.0)	1		1	
CT	350(31.3)	165(29.7)	257(28.7)	0.817(0.663–1.007)	0.058	0.855(0.663–1.103)	0.228
TT	44(3.9)	22(4.0)	29(3.2)	1.300(0.791–2.140)	0.301	0.779(0.423–1.434)	0.423
Additive model				0.934(0.788–1.108)	0.433	0.866(0.704–1.065)	0.173
Dominant model				0.860(0.704–1.050)	0.139	0.841(0.659–1.073)	0.163
Recessive model				1.378(0.842–2.254)	0.202	0.811(0.443–1.484)	0.497
rs11820062							
GG	422(38.0)	181(32.8)	320(35.9)	1		1	
GA	508(45.7)	263(47.6)	388(43.5)	1.265(1.021–1.567)	0.032	0.877(0.676–1.137)	0.321
AA	181(16.3)	108(19.6)	183(20.5)	1.531(1.162–2.016)	**0**.**002**	1.165(0.837–1.622)	0.364
Additive model				1.241(1.086–1.420)	**0**.**002**	1.049(0.894–1.231)	0.558
Dominant model				1.354(1.111–1.650)	**0**.**003**	0.963(0.757–1.226)	0.762
Recessive model				1.346(1.051–1.723)	0.018	1.262(0.940–1.695)	0.121
rs230530							
AA	314(29.2)	132(24.0)	232(26.2)	1		1	
AG	505(47.0)	287(52.1)	441(49.7)	1.324(1.052–1.667)	0.017	0.872(0.656–1.159)	0.344
GG	255(23.7)	132(24.0)	214(24.1)	1.181(0.903–1.544)	0.225	0.845(0.607–1.173)	0.315
Additive model				1.092(0.955–1.250)	0.198	0.919(0.781–1.083)	0.315
Dominant model				1.346(1.092–1.660)	0.005	0.871(0.669–1.133)	0.304
Recessive model				1.010(0.808–1.264)	0.928	0.927(0.710–1.210)	0.578
rs3774963							
GG	442(39.5)	201(36.9)	346(40.0)	1		1	
GC	509(45.5)	265(48.6)	394(45.5)	1.175(0.955–1.448)	0.127	0.891(0.691–1.149)	0.373
CC	167(14.9)	79(14.5)	126(14.5)	0.958(0.954–1.448)	0.774	0.856(0.600–1.220)	0.390
Additive model				1.022(0.890–1.174)	0.756	0.917(0.775–1.085)	0.315
Dominant model				1.072(0.884–1.301)	0.479	0.866(0.684–1.095)	0.229
Recessive model				0.864(0.660–1.131)	0.287	0.900(0.650–1.246)	0.527

Abbreviations: CI, confidence interval; HCV, hepatitis C virus; OR, odds ratio; SNP, single nucleotide polymorphism.

Group A: controls; Group B: spontaneous clearance subjects; Group C: persistent infection patients. Group (B + C): Infected individuals.

^a^The *P* value, OR and 95% CIs of Group (B + C) versus Group A were calculated on the basis of the logistic regression model, adjusted by gender, age and routes of infection.

^b^The *P* value, OR and 95% CIs of Group C versus Group B were calculated on the basis of the logistic regression model, adjusted by gender, age and routes of infection.

Bonferroni correction was applied and the *P* value was adjusted to 0.003125 (0.05/16).

Bold type indicates statistically significant results.

No associations between rs230530, rs1056890, rs3774963 and HCV susceptibility remained statistically significant after Bonferroni correction (all *P* > 0.003).

Further stratified analysis was conducted based on age, gender and routes of infection. Subjects were divided into two subgroups by average age (age < 50 and age ≥ 50) for analysis. As shown in Table 4, the correlation between rs11820062 A allele and the susceptibility to HCV infection was still statistically significant in the young (age < 50), female and blood donors subject subgroups (all *P* < 0.05).

**Table 4:** Stratified analysis of rs11820062 among control, spontaneous clearance and persistent infection groups.

SNPs	Allele	Subgroups	Group (B + C)/Group A	
			OR(95% CI)^a^	*P* ^a^
rs11820062	G/A	Age		
		<50	1.494(1.118–1.995)	**0**.**007**
		≥50	1.237(0.935–1.637)	0.137
		Gender		
		male	1.257(0.912–1.733)	0.162
		female	1.353(1.050–1.743)	**0**.**020**
		Routes of infection		
		HD	1.162(0.791–1.708)	0.442
		Drug use	1.333(0.894–1.988)	0.159
		Blood donation	1.494(1.104–2.021)	**0**.**009**

Abbreviations: CI, confidence interval; HD, hemodialysis; OR, odds ratio.

Group A: controls; Group B: spontaneous clearance subjects; Group C: persistent infection patients. Group (B + C): HCV-infected individuals.

^a^The *P* value, OR and 95% CIs of group (B + C) versus Group A were calculated on the basis of the binary logistic regression model, adjusted by gender, age and routes of infection in dominant model (GG versus [GA + AA] for rs11820062).

Bold type indicates statistically significant results.

### Association between *NF-κB* polymorphisms and spontaneous clearance of HCV infection

No association was observed between the four SNPs (rs1056890, rs11820062, rs230530 and rs3774963) and spontaneous clearance of HCV in our logistic regression analysis (all *P* > 0.003, Table 2), or using additive, dominant, recessive models (all *P* > 0.003, Table 2).

### *In silico* Analysis of SNPs function

**Figure 1:**
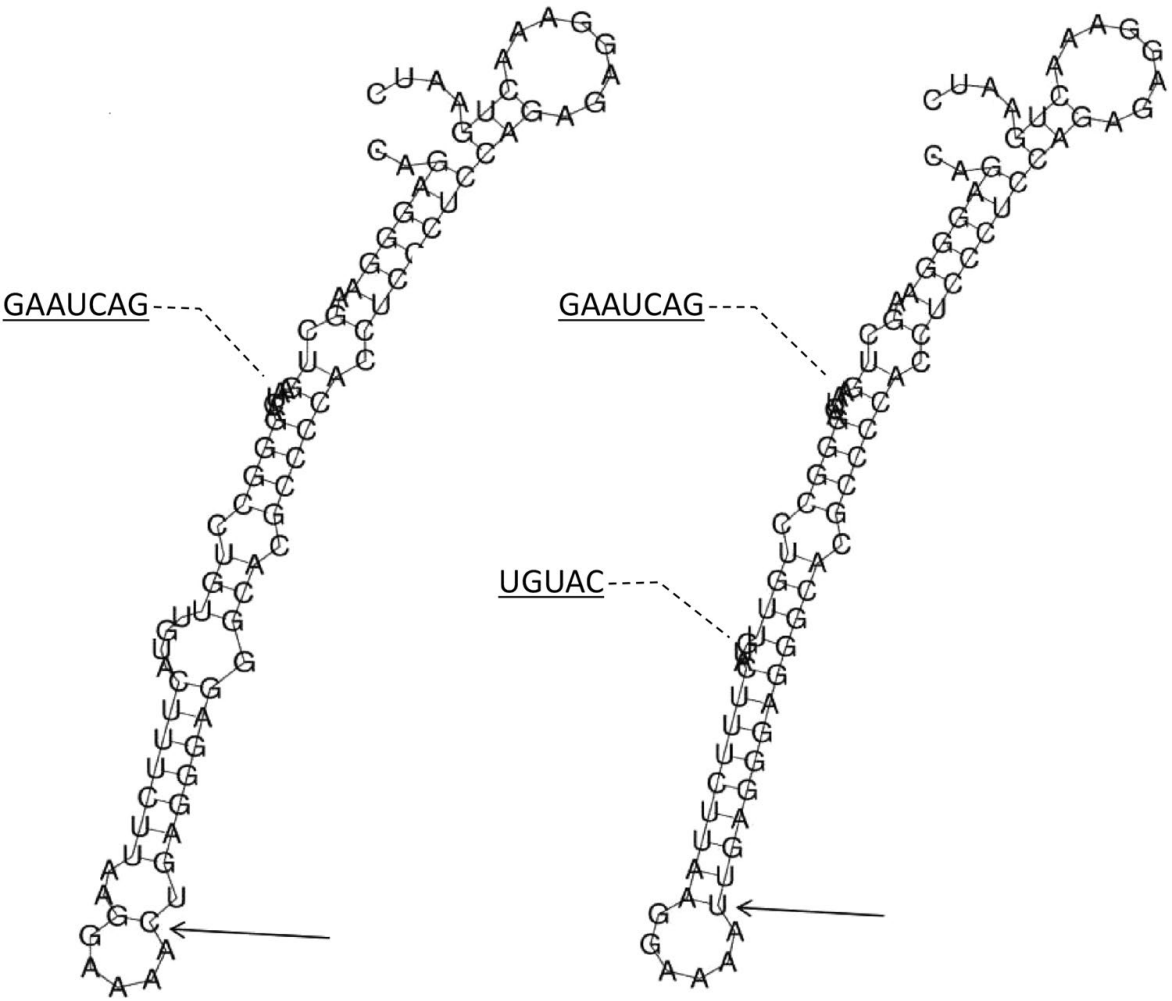
The influence of rs11820062 on mRNA centroid secondary structures of RelA near the 5′ end region. Changes in the local structure were illustrated by the RNAfold Web Server. The arrow indicates the position of the mutation (50 bases upstream and 50 bases downstream from the mutation). The minimum free energy of the mRNA centroid secondary structure (a structure with minimal base pair distance) for wild type and mutant rs11820062 were estimated to be −22.50 kcal/mol (Fig. 1: left figure) and −27.70 kcal/mol (Fig. 1: right figure), respectively. The wild-type and mutant-type sequences are listed below. Underline bold type indicates the overlapping nucleotide letter that are unreadable in figures. The framed type indicates the nucleotide difference between the wild and mutant allele. Wild-type sequence: CAGAGGGAAGCU**GAAUCAG**GGCCUGUUGUACUUUCUUAAGGAAAAUGAGGGAGGGCACGCCCCACCUCCCUCCAGAGAGGAAACUGAAUC Mutant-type sequence: CAGAGGGAAGCU**GAAUCAG**GGCCUGU**UGUAC**UUUCUUAAGGAAAAUGAGGGAGGGCACGCCCCACCUCCCUCCAGAGAGGAAACUGAAUC.

Rs11820062 is located on intron 1 or near the 5′ end of the *RelA* gene (from two different mapping pipelines for rs11820062 on NCBI dbSNP: https://www.ncbi.nlm.nih.gov/snp/), which contains 11 exons and is mapped to chromosome 11q13.1. Using the SNPinfo web server, rs11820062 was predicted to have a TF binding site. Additionally, provided that this SNP is located near the 5′ end of *RelA*, the mutation in this region could also alter the binding of TF and transcriptional regulation. Therefore, the effect of this polymorphism site on the mRNA secondary structure was further analyzed using the RNAfold web server. The local structure changes are shown in Fig. 1. The minimum free energy of the centroid mRNA secondary structure (a structure with minimal base pair distance) for mutant T allele of rs11820062 (corresponded to A allele in this research, −27.70 kcal/mol) was lower than that of wild C allele (corresponded to G allele in this research, −22.50 kcal/mol).

Rs230530 and rs3774963 are located on the *NF-κB1* gene, which contains 24 exons and is mapped to chromosome 4q23-q24. Both SNPs are located in the intron region of *NF-κB1* (rs230530: deep region of intron 3, rs3774963: intron 15). The Human Splicing Finder web server and the SNPinfo web server were used to analyze the effect of the intron variants on splicing or other functions. However, no changes were predicted for either SNPs.

Rs1056890 is located in the 3′-UTR region of the *NF-κB2* gene, which contains 25 exons and is mapped to chromosome 10q24.32. According to the SNPinfo web server, rs1056890 is located within the miRNA binding site and affected gene expression regulation at the post-translational level. However, this SNP was not found to be relevant to HCV infection. The mutation’s influence at this site on the secondary structures of *NF-κB2* 3′-UTR messenger ribonucleic acid (mRNA) was predicted using the RNAfold web server. No differences in the secondary structures between wild-type and mutant type were found (Supplementary Figure 1). The lowest free energy secondary structure for the C allele of rs1056890 is nearly identical to that of the T allele as estimated by MFE (−48.58 kcal/mol versus −46.77 kcal/mol, respectively; Supplementary Figure 1). Additionally, the minimum free energy of the mRNA centroid secondary structures for both mutant type and wild-type of rs1056890 were estimated at −22.20 kcal/mol (Supplementary Figure 1). Therefore, a mutation at this site can not influence the stability and function of the mRNA, *NF-κB2* gene expression or HCV infection.

## Discussion

Our results indicated that rs11820062 A allele was independently associated with the risk effects of HCV infection. Host genetic variation plays a critical role in the pathogenesis and development of HCV infection. Many genes have been reported to affect HCV infection immune response resulting in different disease outcomes. Our previous studies have found that genetic variants of *Toll-like receptor 7* ^22,23^, *interleukin-18* ^24^, *human leukocyte antigen class II* ^25,26^, *vitamin D receptor* ^27^ and *estrogen receptor α* ^28^ genes can affects the susceptibility and resolution of HCV infection. It is believed that NF-κB acts as a switch or a sensor in a wide variety of cellular processes and signaling pathways responsible for inflammatory response to viral infections^9,12^. Polymorphisms in *NF-κB* pathway genes have also been linked to a range of diseases. A growing number of studies suggest that *NF-κB* polymorphisms are related to many immunity-associated diseases, such as hepatocellular carcinoma^29^, colorectal cancer^30^, chronic hepatitis C and liver disease progression^31^, rheumatoid arthritis^32^, asthma^33^ as well as other diseases^13–20^.

In this study, the A allele (mutant type) of rs11820062 (located on intron 1 or near the 5′ end of *RelA*) was found to be linked to susceptibility to HCV infection. RelA protein, also known as p65, is involved in p50/65 complex formation, nuclear translocation and NF-κB activation. Previous research has shown that the SNP rs11820062 confers increased susceptibility to chronic kidney disease^13^ and schizophrenia^14^. Firstly, provided that this SNP is located on intron 1, a multitude of research has confirmed that many regulatory elements, including enhancers, silencers, or other elements that modulate the function of the main upstream promoter, are often within the first intron closest to the 5′ region^34^. This 5′-most intron can stimulate transcription initiation^35^ and is related to mRNA expression levels^34^. Previous *in silico* genotype-gene expression analysis found that this SNP can alter mRNA expression of *RELA* in immortalized B lymphocytes and the binding to androgen receptor^14^. The risk allele (mutant type: A allele in this research) was especially associated with the lower transcriptional activity of *RELA* ^14^. Secondly, according to predictions regarding this SNP location near the 5′ end of *RELA* gene, the minimum free energy of the centroid mRNA secondary structure for mutant type is lower than that of the wild-type. Therefore, the genetic variation of rs11820062 may affect the *RELA* gene transcription process and regulate gene expression, which ultimately affects NF-κB activation and the susceptibility to HCV infection.

In stratification analyses based on age, gender and route of infection, we found that rs11820062 A allele also tracked the risk of HCV susceptibility among young, female, and blood donor subgroups. In general, being young and female are regarded as the common protective factors for hepatitis C because of the youth- and estradiol-related more effective immune response^36,37^. The mechanism of the risk effects among young, female subgroups is not clear in this study. According to *in silico* analysis, a possible cause of the risk associated with rs11820062 T allele showed in young and female subgroups might be related to the disruption of the consensus transcription factor binding sequences (wild allele to risk mutant allele) in the androgen receptor^14^. Furthermore, from 1980 to 1990, blood donors were monetarily compensated, and subsequently numerous donors were found to be infected with HCV. The plasma of some paid donors was separated and collected by plasmapheresis, and the other blood components that contained cross-contamination were returned to the donor. A mandatory HCV screening policy for bloodborne diseases in donated blood was implemented in China in 1993^38^. In this study, the blood donation subjects, especially regarding the unsafe paid blood donors, may result in a high HCV inoculum than that of hemodialysis patients or intravenous drug abuser. Therefore, the subjects in the blood donors subgroup carried the rs11820062 A allele had a significantly increased risk of HCV infection.

Our study was limited because the exact age and time of HCV infection could not be deduced for many patients, which may have affected the evaluation of host immune response and outcomes of HCV infection. Additionally, to reduce the inevitable selection bias in this study, confounding factors were accounted for by matching individuals according to these factors and then adding them as covariates in the logistic regression model.

In conclusion, this study revealed that genetic variants of the *NF-κB* pathway genes (rs11820062 T allele) are associated with an increased risk of HCV susceptibility within a high-risk Chinese population. This finding will inform novel preventive, predictive and therapeutic ideas for HCV infection. However, further functional research on SNP rs11820062 is required.

## Electronic supplementary material

Supplementary Information
