# Supplementary material for: Genetic variations in NF-κB were associated with the susceptibility to hepatitis C virus infection among Chinese high-risk population
Source: Sci Rep. 2018 Jan 8;8:104. doi: 10.1038/s41598-017-18463-y (PMC5758514; doi:10.1038/s41598-017-18463-y)
Supplement: Supplementary file 1 — Supplementary Information [file 41598_2017_18463_MOESM1_ESM.doc]

**Genetic variations in** ***NF-κB* were associated with the susceptibility to hepatitis C virus** **infection among Chinese high-risk population**

Ting Tian^1,2,#^, Jie Wang^3,#^, Peng Huang^1^, Jun Li^4^, Rongbin Yu^1^, Haozhi Fan^1,2^, Xueshan Xia^5^, Yaping Han^4^, Yun Zhang^2^, Ming Yue^4*^

1 Department of Epidemiology and Biostatistics, School of Public Health, Nanjing Medical University, Jiangsu, China;

2 Institute of Epidemiology and Microbiology, Huadong Research Institute for Medicine and Biotechnics, Jiangsu, China;

3 School of Nursing, Nanjing Medical University, Jiangsu, China;

4 Department of Infectious Diseases, The First Affiliated Hospital of Nanjing Medical University, Jiangsu, China;

5 Faculty of Life Science and Technology, Kunming University of Science and Technology, Kunming, China.

# These authors contributed equally to this work.

Correspondence: Dr Ming Yue, Department of Infectious Diseases, The First Affiliated Hospital of Nanjing Medical University, No. 300 Guangzhou Road, Nanjing 210029, Jiangsu, China.

E-mail: [njym08@163.com](mailto:njym08@163.com) or [yueming@njmu.edu.cn](mailto:yueming@njmu.edu.cn)

**
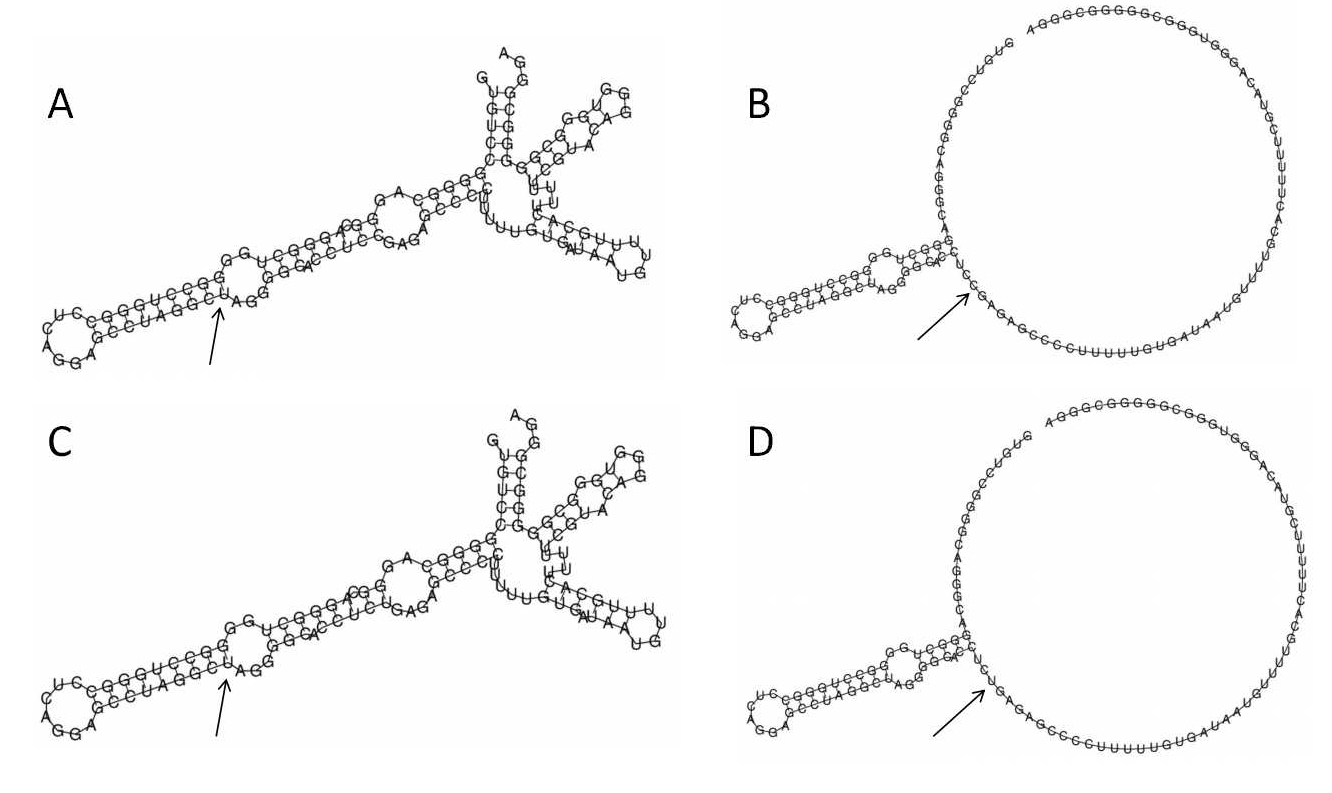
**

**Supplementary figure 1.** The influence of rs1056890 on mRNA minimum free energy (MFE) secondary structures and centroid secondary structures of NF-κB2 3’-UTR. The local structure changes were illustrated by RNAfold Wed Server. The arrows indicate the positions of the mutations (60 bases upstream and 60 bases downstream from the mutation). The minimum free energy of the mRNA MFE secondary structures (a structure contributes a minimum of free energy) for wild type and mutant allele of rs1056890 were estimated at -48.58 kcal/mol (Supplementary figure 1A) and -46.77 kcal/mol (Supplementary figure 1C), respectively. Both of the minimum free energy of the mRNA centroid secondary structure (a structure with minimal base pair distance) for wild type and mutant allele of rs1056890 were estimated at -22.20 kcal/mol (Supplementary figure 1B and Supplementary figure 1D, respectively). The sequences of the wild-type and mutant-type are listed as below. The framed type indicates the wide/mutant allele. The framed type indicates the nucleotide difference between the wild and mutant allele.

Wild-type sequence:

GUGUCCGGGGCAGGGCAGGGCUGGGGCCUGGGCCUCAGGAGCCUAGGCUAGGGGCACCUCCGAGAGCCCCUUUUUGUGAUAAUGUUUUGCACUUUUUCGUACAGGGUGGGCGGGGGCGGGA

Mutant-type sequence:

GUGUCCGGGGCAGGGCAGGGCUGGGGCCUGGGCCUCAGGAGCCUAGGCUAGGGGCACCUCUGAGAGCCCCUUUUUGUGAUAAUGUUUUGCACUUUUUCGUACAGGGUGGGCGGGGGCGGGA
